# Supplementary material for: Quantifying 35 transcripts in a single tube: model-based calibration of the GeXP multiplex RT-PCR assay
Source: BMC Biotechnol. 2021 Apr 14;21:29. doi: 10.1186/s12896-021-00689-4 (PMC8048187; doi:10.1186/s12896-021-00689-4)
Supplement: Supplementary file 1 — Additional file 1. [file 12896_2021_689_MOESM1_ESM.pdf]

Quantifying 35 transcripts in a single tube: Model-based calibration of the GeXP RT-PCR assay

Pauline Marquardt, Britta Werthmann, Viktoria Rätzel,
Markus Haas and Wolfgang Marwan

Supplementary Information

Supplementary Figures and Legends .... p. 2 of this pdf

Protocol for the installation of Perl scripts for the GeXP data analysis workflow .... p. 8 of this pdf

Additional supplementary files:

Additional file 2 Table S1

Additional file 3 Figure S6

are provided with the electronic version of the main article.

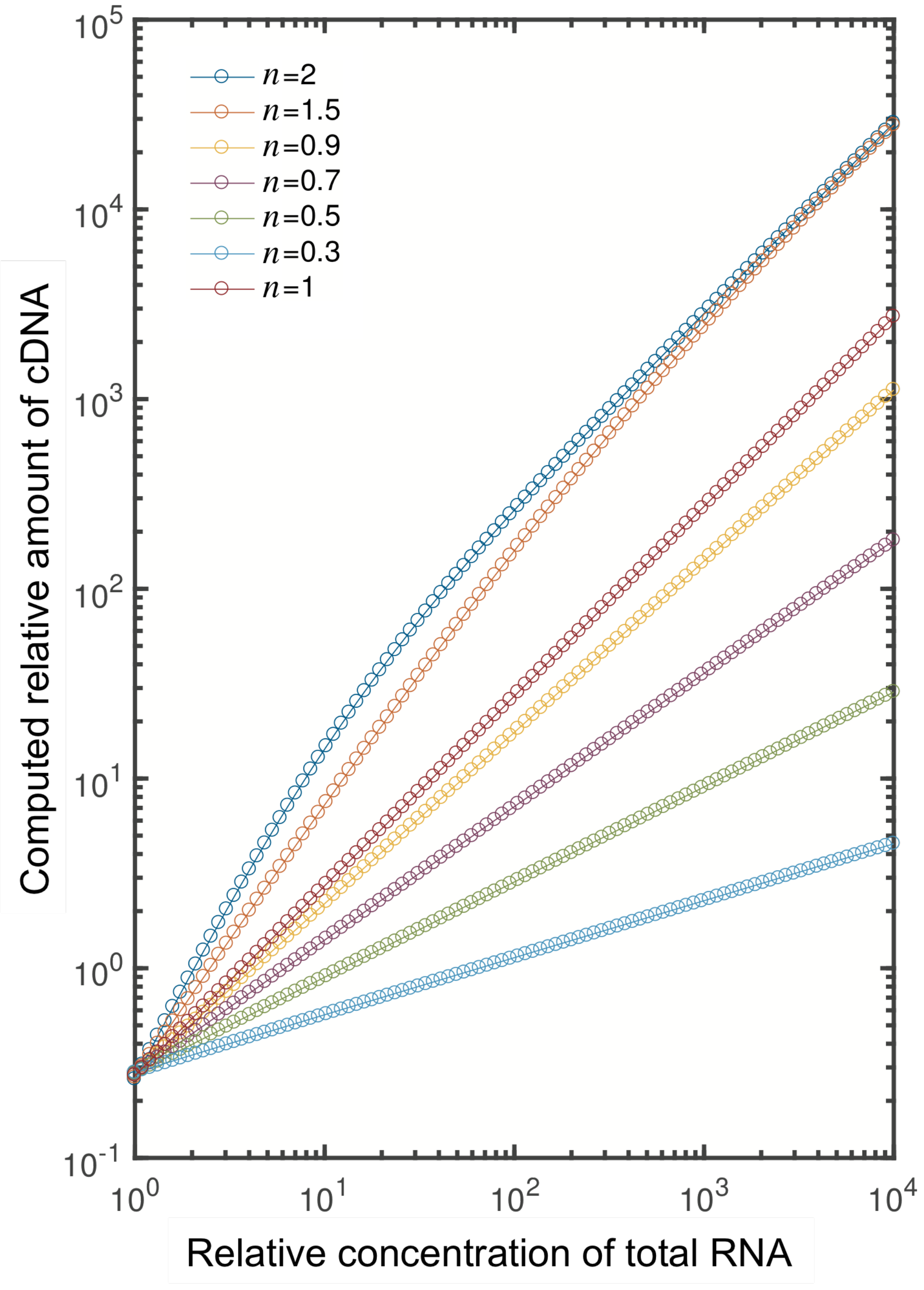

Figure S1. Calibration curves as predicted by the kinetic model of the GeXP RT-PCR reaction for different empirical reaction orders n as indicated in the inset. The relative concentration of cDNA synthesized during the RT reaction was computed as a function of total RNA concentration according to eqns. (11) and (12). The model predicts approxiamtely linear calibration curves for reaction orders of $n \leq 1$ in the double logarithmic plot.

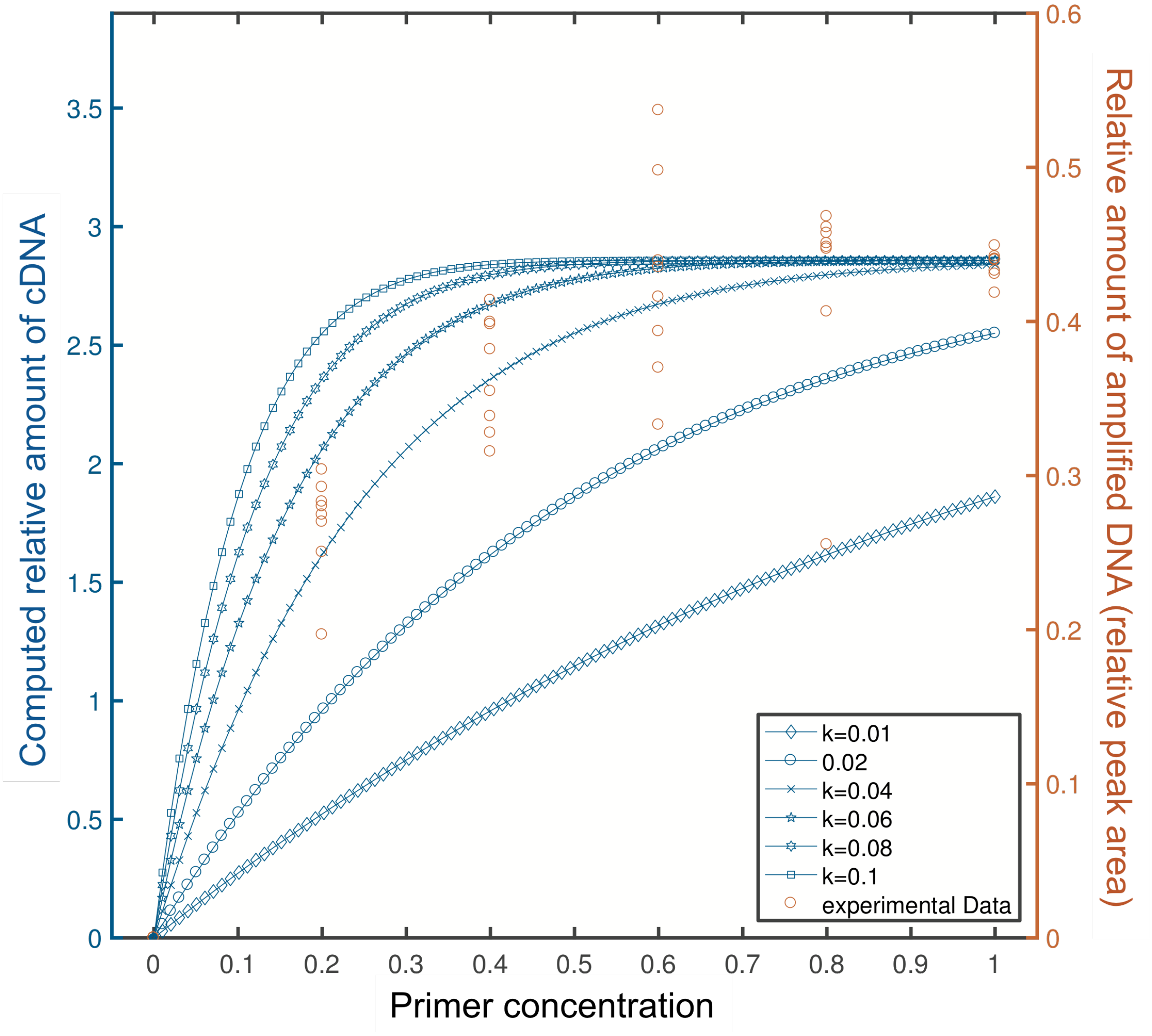

Figure S2. Concentration of an amplified cDNA fragment as a function of the concentration of the *pcnA*-gene-specific reverse primer used for the RT reaction. Experimental values are represented by brown symbols. Blue symbols represent computational results for a reaction order of n=0.9 as obtained from eqn. (12) for different values of the primer-specific rate constant k'. The relative primer concentration of 1.0 corresponding to 50 nM was used in the RT reaction of the standard GeXP experiments.

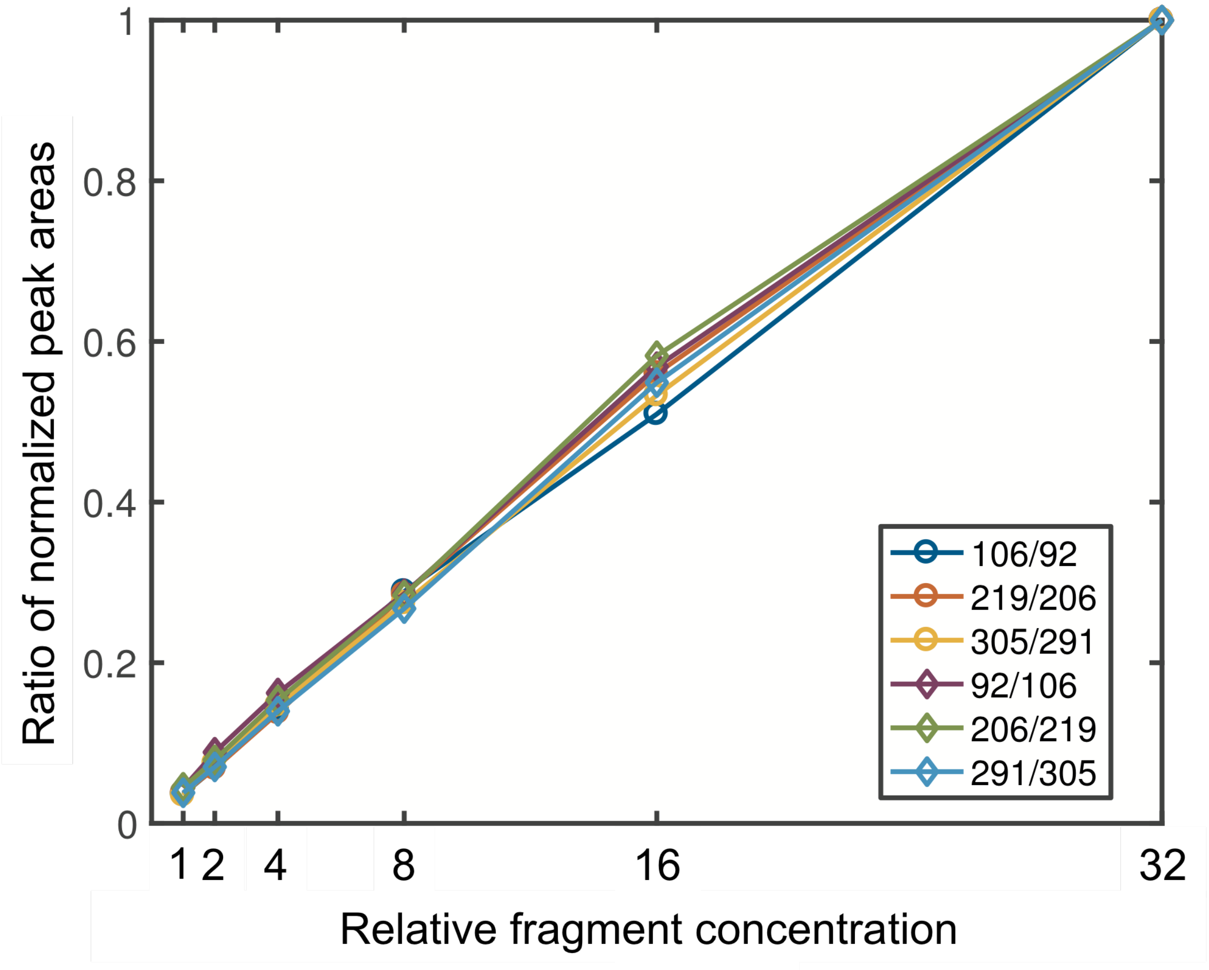

Figure S3. Calibration of fluorescent DNA fragment quantification by the CEQ 8800 Genetic Analysis System. Two cocktails, each containing three fluorescently labelled PCR products of different lengths (as amplified from phage lambda DNA) were prepared (cocktail #1: 92 nt, 206 nt, 291 nt; cocktail #2: 106 nt, 219 nt, 305 nt). The cocktails were mixed at different relative fragment concentrations, separated on the CEQ 8800 Genetic Analysis System, and the normalized peak area (NPA) for each fragment determined using the custom software delivered with the system. The ratio of the normalized peak areas (NPAs) measured for pairs of fragments (as indicated in the inset) were plotted against the relative concentration of cocktail #1 or cocktail #2, respectively. Values fitted to a straight line with a slope of 1, confirming that the peak area as determined by the software was proportional to the amount of DNA fragment subjected to capillary electrophoresis.

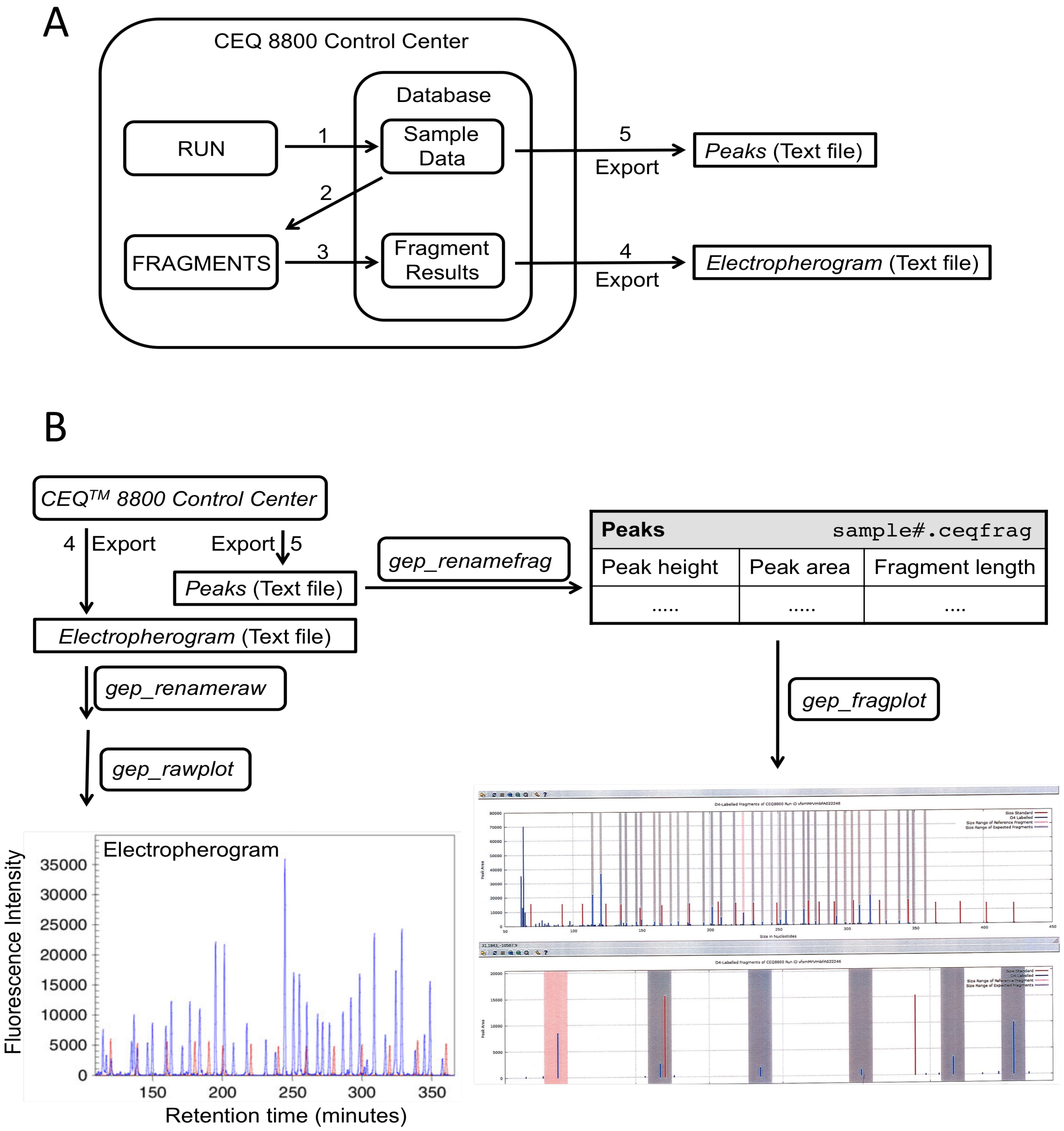

Figure S4. Electrophoresis and signal processing. (A) A DNA ladder of fragments of known size is added to each sample of amplified cDNAs and fragments are separated electrophoretically on a CEQ 8800 analyser. The CEQ^{TM} 8800 software (Control Center) saves the electropherogram as Sample Data in the database (step 1). After the run is completed, the operator calls the *FRAGMENTS* module of the Control Center to generate the Fragment Results, a table which assigns the peak height, the peak area, and the calculated fragment length to each peak detected above threshold (step 2) which are also stored in the database of the Control Center (step 3). All peaks for which the peak height is smaller than 0.5% of the height of the second largest peak are considered to be below threshold. The cutoff value of 0.5% as used in this study is to be specified by the user of the CEQ^{TM} 8800 Control Center, is kept constant for all experiments, and defines the detection limit for each peak. The *Peaks* (Fragment Results, consisting of peak height, peak area, and fragment

length) and the *Electropherogram* (Sample Data, fluorescence intensity versus retention time) of each sample are exported from the database of the CEQ8800 Control Center in the form of tables as tab-separated text files (steps 4 and 5, respectively). The *Peaks* file is lateron used for calibration or fragment quantification (see Figure S5). (B) For convenient analysis, the *Electropherogram* and *Peaks* files may be transferred to a separate personal computer for further data processing. The *Electropherogram* file extension is renamed to change the file extension to .ceqraw and displayed in Gnuplot by running the scripts *gep_renameraw* and *gep_rawplot*, respectively. The graphical representation of each electropherogram (panel lower left; peaks of cDNA fragments in blue, peaks of the DNA size standard in red) is visually inspected to assess the quality of the separation run before the data are further numerically analyzed. The *Peaks* file extension is renamed by the script *gep_renamefrag* as well in order to specify the file type. The panel lower right is generated by the script *gep_fragplot*. It graphically displays the detected peaks in relation to the size ranges of the expected fragments (blue) and the size ranges of the reference fragments (of the DNA size standard; red) which both are used to automatically assign the detected peaks to the respective fragments. With the help of this plot one can assess the quality of each separation.

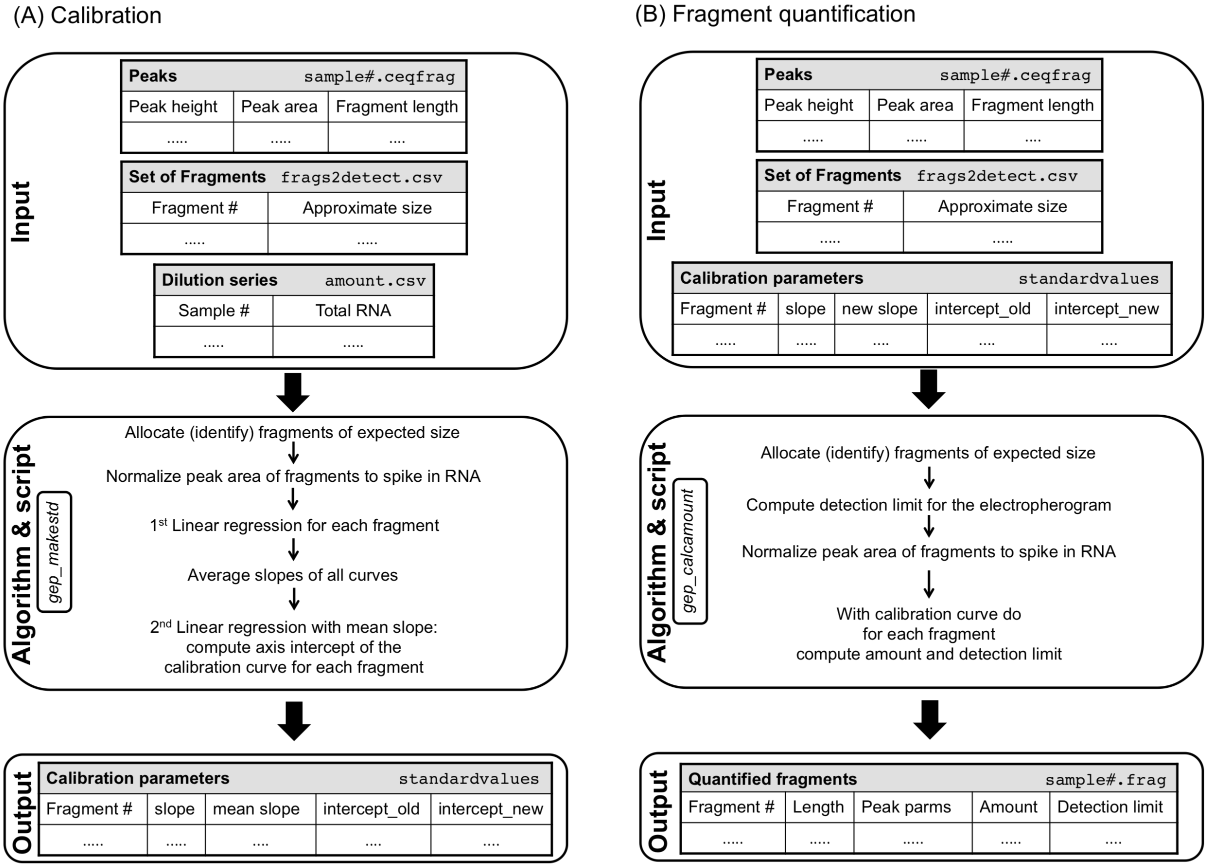

Figure S5. Script-based evaluation of Peaks. (A) Calibration. A calibration curve is recorded for each of the fragments to be analysed. To generate the calibration curves, an RNA sample that contains the RNAs of interest at intermediate concentration (see legend to Fig. 2 for details) is serially diluted and the diluted samples with their total RNA concentrations are entered to give the table *Dilution series* that together with the list of expected fragments (*Fragments*) serve as an input read by the script *gep_makestd*. For all fragments to be quantified, this script identifies fragment-specific peaks by assigning the largest peak from the *list of fragments* file that has a measured fragment length of ±0.8 nt of the expected approximate size as listed in the *Fragments* file for the respective fragment. The script then normalizes the peak area of each fragment to the peak area measured for the spike-in RNA. With these values the script computes for each fragment a calibration curve by performing a linear regression of the log (normalized peak area) versus log (total RNA concentration) values (1st linear regression). The slopes obtained for all expected gene-specific fragments in the 1st linear regression are averaged and the data points for each gene-specific fragment are fitted to a straight line by a 2nd linear regression that computes the intercept obtained for the average slope. The resulting curve fit parameters of each gene-specific fragment to be analysed, are stored in the *Calibration curves* file. The entries in the *Calibration curves* file are inspected line-wise for consistency of slope and intercept values obtained by the 1st and the 2nd linear regression. (B) Fragment quantification. The curve fit parameters (mean slope and intercept obtained by the 2nd linear regression) of the calibration curves (as contained in the file *Calibration curves*) are used to quantify gene-specific fragments by evaluating *Peaks* files obtained by analysing experimental samples. For each gene-specific fragment in each sample, the script *gep_calcamount* calculates its relative amount and the corresponding detection limit. The detection limit may be different for each gene-specific fragment and differ between electrophoretic separations. It is estimated taking the 0.5% value of the height of the

second largest peak in the electrophoretic separation of the analysed sample and the parameters (mean slope and intercept) of the 2nd linear regression of the valid calibration curve obtained for each respective fragment. For download of the relevant scripts and example data files see Supplementary Information.

Legends to Additional Supplementary Files

Figure S6. Comparative display of single cell gene expression time series in response to a sporulation-inducing far-red light stimulus, first measurement and technical replicate. The figure which is provided Additional file 3 in the online version of this article shows a full-length version of Fig. 5 with the values for the transcripts of all 35 genes displayed. For further details see legend to Fig. 5. Because of its size, the figure is best inspected with the help of a pdf viewer with appropriately adjusted zoom factor.

Table S1. Sequences and concentrations of primers used for GeXP-RT-PCR. The Table is provided in the form of an Excel sheet and is provided as part of the Supplementary Material to the online version of this paper.

Protocol for the installation of Perl scripts for the GeXP data analysis workflow

Installation under Linux

To run the software on a Linux computer, retrieve the files from https://github.com/markushaas/genexpro4ceq8800 and install them by following the install instructions that are provided together with the files.

Installation under Mac OS X or Windows^R

For running the Perl scripts under Mac OS X or Windows^R operating systems (os), we have set up a virtual Linux machine (vm), a package, in which the Linux operating system and all files necessary for the GeXP data analysis workflow (Perl scripts, example files, and ReadMe) are put in place. Download from github is therefore not necessary. Installation of the virtual machine can be easily performed by going step by step through the following protocol:

- Install "VirtualBox" from Oracle (https://www.virtualbox.org/).
- Download the vm image file we have prepared by clicking on the following link http://www.regulationsbiologie.ovgu.de/Downloads.html or by copying the link into the address bar of your browser window.
- Open "VirtualBox" and select "Import Appliance..." from the "File" menu, choose the vm image file "gep_vm.ova" you have downloaded and follow the instructions of the „VirtualBox“ dialogue.
- To share a folder with your host os (for shutteling files between your os and the virtual machine), create a folder with the name ".virtualboxsharedfolder". If you are using a Windows® pc you may need to create the folder within a terminal window of your os as the file manager does not accept names

beginning with a "." so execute cmd.exe and enter "mkdir .virtualboxsharedfolder".

- Right click on the "gep_vm" item that shows up in the VirtualBox window and select "Settings" and therein "Shared Folders". Click on the "Add new shared folder" icon, select the folder ".virtualboxsharedfolder", and enable "Automount". Files in this folder can be accessed by both, your host and the operating system run by the virtual machine.
- Start the vm. Depending on the BIOS installed on your computer, you may have to enable "Intel® Virtualization Technology" in the BIOS settings in order to allow you to use a vm on your pc. Refer to the manual of your hardware if necessary.
- After booting the vm open the "Introduction" file (by double clicking the icon) on the Desktop of the vm to get some help with the GeXP data analysis workflow. To adapt the resolution of the virtual screen, adjust the settings in the "view" menu of the virtual machine window.
- In case of problems with the shared folder or the screen resolution that might occur under the particular os you are working with, refer to the internet to get help.
