# Supplementary material for: Quantifying 35 transcripts in a single tube: model-based calibration of the GeXP multiplex RT-PCR assay
Source: BMC Biotechnol. 2021 Apr 14;21:29. doi: 10.1186/s12896-021-00689-4 (PMC8048187; doi:10.1186/s12896-021-00689-4)
Supplement: Supplementary file 2 — Additional file 2 Table S1. Sequences and concentrations of primers used for GeXP-RT-PCR. [file 12896_2021_689_MOESM2_ESM.pdf]

Transcript ID	Gene	Similarity	Organism	UniProtKB	% Query Coverage	E-value	RW-Primer ID	Sequence of reverse primers	FW-Primer ID	Sequence of forward primers	RW-Primer concentration in RT reaction [nM]	Length of amplified fragments
Phypoly_transcript_07700	*anxA*	Annexin VII	*Mus musculus*	Q07076	39.157	1.04E-32	#1690	GTACGACTCACTATAGGGACTCTTGGCAAAGAAGGCTTG	#1689	AGGTGACACTATAGAATATGAGAAAGCCGAAGAGGATG	50.000	292
Phypoly_transcript_06541	*ardA*	Actin, plasmodial isoform	*Physarum polycephalum*	P02576	100	0	#1654	GTACGACTCACTATAGGGAAGGTGTGGTGCCAGATTTTC	#1653	AGGTGACACTATAGAATATGGTGTGATGGTTGGTATGG	0.098	183
Phypoly_transcript_11686	*arpA*	Probable basic-leucine zipper transcription factor G	*Dictyostelium discoideum*	Q54RZ9	44.068	1.15E-22	#1684	GTACGACTCACTATAGGGACCTTCAAGATCCCCAACCTT	#1683	AGGTGACACTATAGAATAATCGCACAACCCACTACTCC	50.000	272
Phypoly_transcript_05813	*cdcA*	Caltractin isoform 1	*Homo sapiens*	P41208	59.756	1.26E-32	#1652	GTACGACTCACTATAGGGACTGACGGATGAGGAGCTTTC	#1651	AGGTGACACTATAGAATAGATATGTCAACAATCGCCCC	50.000	177
Phypoly_transcript_06284	*cudA*	Putative transcriptional regulator cudA	*Dictyostelium discoideum*	O00841	43.478	3.96E-28	#1696	GTACGACTCACTATAGGGAGGGTTTGGAGATGACCTTGA	#1695	AGGTGACACTATAGAATAGACCTCAACCACATTGCCTT	12.500	309
Phypoly_transcript_01287	*damA*	DNA damage-binding protein 1	*Oryza sativa Japonica Group*	Q6L4S0	61.892	0	#1644	GTACGACTCACTATAGGGATTGTCGTGTTTCAGGTCCAA	#1643	AGGTGACACTATAGAATAGGAGGTTTTACCCACGAACA	0.390	149
Phypoly_transcript_03245	*dspA*	DNase TatD	*Pantoea vagans*	E1SKR8	28.986	9.63E-31	#1670	GTACGACTCACTATAGGGATTTTTGATCACATTTCGGGC	#1669	AGGTGACACTATAGAATAGTGGAGAGCCTGGAGTGGTA	12.500	231
Phypoly_transcript_06735	*ehdA*	EH domain-containing protein 1	*Bos taurus*	Q5E9R3	41.315	9.57E-130	#1646	GTACGACTCACTATAGGGATTCAAGCAGGTCACAAGGTG	#1645	AGGTGACACTATAGAATAATCGAATCAGGTCTCGCTGT	3.130	159
Phypoly_transcript_16748	*gapA*	ADP-ribosylation factor GTPase-activating protein	*Arabidopsis thaliana*	O82171	35.22	2.61E-24	#1676	GTACGACTCACTATAGGGATTTATGGGCAAAGAATTCGC	#1675	AGGTGACACTATAGAATATGAGTTGTTTTGACTGCGGA	50.000	251
Phypoly_transcript_17984	*hcpA*	Anti-silencing function protein 1 homolog A	*Bos taurus*	Q2KIG1	52.88	8.27E-64	#1706	GTACGACTCACTATAGGGATCATCTCTTCCGTCATGCTG	#1705	AGGTGACACTATAGAATATTACTGGGGGTTACTGCGAT	50.000	348
Phypoly_transcript_18509	*hstA*	Probable histone H2B 4	*Caenorhabditis elegans*	Q27876	59.559	2.41E-37	#1658	GTACGACTCACTATAGGGAAAGCTCACCAGGAAGGATCA	#1657	AGGTGACACTATAGAATATCTCCAAGAAAGCGATGGTC	1.560	195
Phypoly_transcript_16372	*ligA*	Checkpoint protein hus1 homolog	*Dictyostelium discoideum*	Q54NC0	41.241	7.24E-61	#1640	GTACGACTCACTATAGGGACTGTAAAGGAACCGGGAAAA	#1639	AGGTGACACTATAGAATAAACAAACTTGGACACCCAGC	6.250	139
Phypoly_transcript_00697	*meiB*	Protein MEI2-like 5	*Arabidopsis thaliana*	Q8VWF5	73.545	6.34E-80	#1678	GTACGACTCACTATAGGGATCTGGAAGTGGGCTATGAGG	#1677	AGGTGACACTATAGAATACCACAAGGGCACCTATGACT	50.000	255
Phypoly_transcript_18969	*nhpA*	Non-histone chromosomal protein 6	*Debaryomyces hansenii*	Q6BRB4	55.224	4.57E-15	#1634	GTACGACTCACTATAGGGATTATGCGAGAGCAGCCTTTT	#1633	AGGTGACACTATAGAATACCACTGCTGCAGACAAGAAG	3.130	120
Phypoly_transcript_00387	*pakA*	Serine/threonine-protein kinase *pakA*	*Dictyostelium discoideum*	Q55D99	41.085	1.96E-87	#1650	GTACGACTCACTATAGGGATTCCAAAATCGGCTAGCAAC	#1649	AGGTGACACTATAGAATAGATTGCCTACGTTTGTGCCT	50.000	171
Phypoly_transcript_13303	*pcnA*	Proliferating cell nuclear antigen	*Brassica napus*	Q43124	55.484	1.27E-81	#1680	GTACGACTCACTATAGGGATGTGTCTCCGTTGTCCTCTG	#1679	AGGTGACACTATAGAATAATCAAGCCAACTTCGACTGC	50.000	260
Phypoly_transcript_00857	*pikB*	Phosphatidylinositol 3-kinase 2	*Dictyostelium discoideum*	P54674	68.809	0	#1672	GTACGACTCACTATAGGGATGCGCAAATAGTTGATGTCC	#1671	AGGTGACACTATAGAATAGCCATTTATTTTCACAGCCC	12.500	237
Phypoly_transcript_01882	*pikC*	Phosphatidylinositol 4-kinase beta	*Sorex araneus*	B3EX61	45.667	6.23E-76	#1700	GTACGACTCACTATAGGGAGAAATCATACGTGCGAGTGG	#1699	AGGTGACACTATAGAATAAAGCTCACGCAGGAGTTCAT	50.000	328
Phypoly_transcript_02833	*pksA*	Serine/threonine-protein kinase phg2	*Dictyostelium discoideum*	Q54QQ1	50.68	1.34E-93	#1638	GTACGACTCACTATAGGGAGGGTGCGTAGTATCTCCCTG	#1637	AGGTGACACTATAGAATAAAGGCATGTTATTTACGCCG	12.500	135
Phypoly_transcript_02552	*pldA*	Phosphatidylinositol-glycan-specific phospholipase D	*Rattus norvegicus*	Q8R2H5	32.237	2.26E-71	#1688	GTACGACTCACTATAGGGAACAAACTGCGGCAGAGAGAT	#1687	AGGTGACACTATAGAATATGGTGAGTCAGCATACCCAA	6.250	286
Phypoly_transcript_04506	*pldB*	Phosphatidylinositol-glycan-specific phospholipase D	*Homo sapiens*	P80108	28.774	4.91E-49	#1702	GTACGACTCACTATAGGGATATCACCGCCTAAATCTGCC	#1701	AGGTGACACTATAGAATACTGTTTTTCCTGACTGGGGA	50.000	338
Phypoly_transcript_02197	*pldC*	Phosphatidylcholine-hydrolyzing phospholipase D1	*Schizosaccharomyces pombe*	Q09706	31.068	7.83E-55	#1698	GTACGACTCACTATAGGGACTTCCCCATATAAAGGCGCT	#1697	AGGTGACACTATAGAATATTGTGGGATCTGGGAACATT	50.000	317
Phypoly_transcript_23026	*pptA*	Probable inactive purple acid phosphatase 29	*Arabidopsis thaliana*	Q9FMK9	46.269	1.10E-09	#1692	GTACGACTCACTATAGGGAGGTTGACTGGTGGATGGAAC	#1691	AGGTGACACTATAGAATAAAACACAGGACTTTTTGCCG	50.000	298
Phypoly_transcript_08298	*pptB*	Probable protein phosphatase 2C 34	*Oryza sativa Japonica Group*	Q94H98	30.208	1.11E-28	#1662	GTACGACTCACTATAGGGATAGTGCTAGGGGCCAATGAG	#1661	AGGTGACACTATAGAATAGCCACCGATTAAATATCCCC	50.000	208
Phypoly_transcript_16094	*psgA*	*Physarum*-specific gene A	*Physarum polycephalum*				#1660	GTACGACTCACTATAGGGATTTACTTGCGGTGTGGAACC	#1659	AGGTGACACTATAGAATAAACACATGATTGGGTGAGCA	1.560	201
Phypoly_transcript_00670	*pumA*	Pumilio homolog 1	*Arabidopsis thaliana*	Q9ZW07	61.281	1.91E-138	#1648	GTACGACTCACTATAGGGACGATAATCTTCTCGATCCGC	#1647	AGGTGACACTATAGAATAAAAAATGCTCGATTTGGTCG	0.390	163
Phypoly_transcript_11692	*pwiA*	Piwi-like protein Ago3	*Bombyx mori*	A9ZSZ2	30.672	8.11E-37	#1694	GTACGACTCACTATAGGGAAGGAAAGCCATTTTGTGTGC	#1693	AGGTGACACTATAGAATAAGGTTATGATGCCGTGAACC	50.000	303
Phypoly_transcript_17606	*ralA*	Circularly permutated Ras protein 1	*Dictyostelium discoideum*	Q75J93	39.161	7.24E-21	#1642	GTACGACTCACTATAGGGACACATTCCATGTTTTTGCCA	#1641	AGGTGACACTATAGAATACGAAAAACGAACACCCTTGT	0.391	146
Phypoly_transcript_09675	*rasA*	Ras-like GTP-binding protein YPT1	*Phytophthora infestans*	Q01890	43.931	3.66E-43	#1686	GTACGACTCACTATAGGGACGGAATTGCTGAGATTGGAT	#1685	AGGTGACACTATAGAATATGGGAACAAAACGGACTCTC	3.130	276
Phypoly_transcript_12613	*rgsA*	Regulator of G-protein signaling 2	*Mus musculus*	O08849	41.176	0.048	#1708	GTACGACTCACTATAGGGAGGTGCTGCAGACATCCTGTA	#1707	AGGTGACACTATAGAATAGGTGCCCTTAAGCAAATCAA	50.000	357
Phypoly_transcript_02603	*ribA*	Poly(ADP-ribose) glycohydrolase	*Drosophila melanogaster*	O46043	42.67	4.66E-95	#1632	GTACGACTCACTATAGGGAATCGCACCTTGAACTTGGTT	#1631	AGGTGACACTATAGAATAGACTGCCAGAAGAAAGGACG	0.025	114
Phypoly_transcript_01309	*ribB*	Poly(ADP-ribose) glycohydrolase	*Oryza sativa Japonica Group*	Q9AV81	50.313	6.02E-146	#1682	GTACGACTCACTATAGGGATTCTTGGTCACGACAGCTTG	#1681	AGGTGACACTATAGAATAGTGGCTGGTGTGTCTTCCTT	6.250	268
Phypoly_transcript_06597	*spiA*	Polyribonucleotide nucleotidyltransferase	*Staphylococcus haemolyticus*	Q4L5X7	23.932	0.13	#1704	GTACGACTCACTATAGGGATGTTGACCCAATAGCATCCA	#1703	AGGTGACACTATAGAATAATGCCGATTTGTTGGAGAAG	50.000	344
Phypoly_transcript_02655	*tspA*	Tumor suppressor p53-binding protein 1 homolog	*Caenorhabditis elegans*	Q7JKP6	33.803	1.16E-05	#1666	GTACGACTCACTATAGGGACCGTGAGTTCCGAGTCCTAA	#1665	AGGTGACACTATAGAATAAATCACAGCAAACGCCAATA	50.000	217
Phypoly_transcript_03260	*uchA*	Secretory immunoglobulin A-binding protein EsiB	*Escherichia coli*	A0A0H2VDN9	33.562	9.47E-14	#1674	GTACGACTCACTATAGGGATGCTCTTTTGCCTTCGAAAT	#1673	AGGTGACACTATAGAATAGATTTGGACAACCTTGAGCC	12.500	245
