# Supplementary material for: Quantifying 35 transcripts in a single tube: model-based calibration of the GeXP multiplex RT-PCR assay
Source: BMC Biotechnol. 2021 Apr 14;21:29. doi: 10.1186/s12896-021-00689-4 (PMC8048187; doi:10.1186/s12896-021-00689-4)
Supplement: Supplementary file 3 — Additional file 3 Fig. S6. Comparative display of single cell gene expression time series. The figure legend is provided as part of Additional file 1. [file 12896_2021_689_MOESM3_ESM.pdf]

		6d, 5 min FR				6d, 15 min FR									7d, 20 min FR				7d, 30 min FR								
	Time (h)	P1	P2	P3	P4	P5	P6	P7	P8	P9	P10	P11	P12	P13	P14	P15	P16	P17	P18	P19	P20	P21	P22	P23	P24	P25	P26
psgA	0	100	100	100	100	100	88	80	100	100	100	100	100	100	100	100	100	100	100	100	100	100	100	100	100	100	73
	2	58	39	85	65	30	100	48	25	99	79	48	78	73	29	26	2	46	31	12	24	45	6	14	56	84	100
	6	10	27	68	31	19	3	100	32	39	15	16	64	11	28	1	23	14	35	4	21	32	12	6	45	50	0
	8	4	6	18	27	2	6	6	5	6	14	9	2	6	7	1	6	3	6	3	37	32	10	1	29	49	19
	11	14	12	7	1	1	1	3	3	7	7	0	21	8	2	2	1	1	1	4	0	9	12	2	13	3	10
pldC	Time (h)	P1	P2	P3	P4	P5	P6	P7	P8	P9	P10	P11	P12	P13	P14	P15	P16	P17	P18	P19	P20	P21	P22	P23	P24	P25	P26
	0	100	33	100	100	32	47	100	100	62	100	100	26	24	100	100	100	100	100	100	100	51	100	100	100	55	100
	2	15	58	75	28	100	100	43	1	43	22	34	100	30	42	99	2	12	84	38	18	24	23	33	87	3	19
	6	2	27	4	14	11	6	3	1	27	2	2	42	30	15	0	3	39	44	29	13	18	1	4	61	19	3
	8	1	100	1	6	6	82	1	0	43	3	1	3	28	8	1	2	5	53	4	15	100	1	2	28	100	3
	11	2	49	2	1	8	12	1	0	100	9	1	14	100	1	9	1	4	20	1	0	25	5	1	18	1	6
pptB	Time (h)	P1	P2	P3	P4	P5	P6	P7	P8	P9	P10	P11	P12	P13	P14	P15	P16	P17	P18	P19	P20	P21	P22	P23	P24	P25	P26
	0	100	10	23	70	100	100	94	100	73	40	45	21	100	100	100	100	100	100	83	79	52	80	100	22	26	100
	2	25	44	45	25	34	54	100	65	40	25	78	12	38	71	41	83	60	37	30	33	33	86	57	79	44	87
	6	18	56	35	100	37	30	28	28	100	30	100	94	22	42	9	40	34	80	44	28	100	100	71	100	48	0
	8	11	54	17	53	33	18	33	75	24	53	99	87	14	37	9	16	24	41	51	100	64	86	58	45	100	89
	11	7	100	100	14	32	12	46	84	47	100	77	100	15	11	2	6	6	30	100	38	54	41	26	70	16	59
pldB	Time (h)	P1	P2	P3	P4	P5	P6	P7	P8	P9	P10	P11	P12	P13	P14	P15	P16	P17	P18	P19	P20	P21	P22	P23	P24	P25	P26
	0	100	100	100	17	33	100	60	100	81	100	100	24	12	100	100	89	20	100	100	100	24	65	76	14	30	48
	2	17	0	36	9	39	28	100	38	100	68	50	14	25	58	12	100	32	90	57	44	33	73	100	50	21	76
	6	13	89	63	100	100	12	83	20	56	68	38	100	0	52	16	8	100	82	42	38	100	100	59	100	100	100
	8	1	71	4	17	7	0	3	2	12	43	8	16	0	4	17	4	5	25	38	8	38	75	29	41	55	17
	11	2	52	3	1	5	3	2	1	82	20	2	17	100	1	15	0	20	30	26	22	88	52	59	82	25	1
pikB	Time (h)	P1	P2	P3	P4	P5	P6	P7	P8	P9	P10	P11	P12	P13	P14	P15	P16	P17	P18	P19	P20	P21	P22	P23	P24	P25	P26
	0	100	36	100	100	100	100	100	100	100	64	100	87	100	100	100	100	100	100	100	96	100	100	100	100	100	100
	2	29	6	22	6	15	98	75	8	96	29	24	46	87	56	50	26	47	14	25	95	0	80	34	100	52	36
	6	12	59	31	12	21	47	83	23	78	26	63	100	47	8	19	25	11	3	45	100	31	30	10	72	59	18
	8	13	65	23	9	14	9	25	4	43	100	35	34	57	2	2	14	5	3	23	51	28	33	3	64	51	47
	11	9	100	22	4	17	57	29	19	44	60	20	31	86	3	0	7	2	4	0	23	31	24	0	64	15	45
ralA	Time (h)	P1	P2	P3	P4	P5	P6	P7	P8	P9	P10	P11	P12	P13	P14	P15	P16	P17	P18	P19	P20	P21	P22	P23	P24	P25	P26
	0	100	20	100	100	85	100	77	100	100	100	100	100	100	100	100	71	100	100	100	100	92	100	100	3	100	100
	2	26	57	94	47	100	27	100	26	96	80	92	67	84	39	47	29	100	31	46	11	32	51	31	30	76	14
	6	10	46	61	68	55	16	21	23	39	68	77	17	46	33	25	100	9	28	33	7	54	45	8	56	46	22
	8	7	100	48	15	24	9	11	21	17	57	49	29	15	16	8	59	5	31	17	11	100	28	11	100	21	28
	11	2	76	45	3	14	8	23	21	8	28	20	30	10	2	9	4	1	10	15	6	58	74	13	85	19	20
nhpA	Time (h)	P1	P2	P3	P4	P5	P6	P7	P8	P9	P10	P11	P12	P13	P14	P15	P16	P17	P18	P19	P20	P21	P22	P23	P24	P25	P26
	0	100	22	100	100	91	92	35	100	100	100	100	49	76	100	100	93	100	100	91	100	71	13	100	100	1	100
	2	31	51	85	34	100	100	100	18	88	53	62	56	100	53	12	61	69	4	100	4	64	52	26	56	6	46
	6	8	93	22	48	52	18	73	19	54	29	40	67	25	45	2	100	19	20	44	13	46	16	84	48	47	2
	8	13	100	20	43	9	9	23	14	22	56	22	11	23	26	3	20	0	11	63	23	34	6	34	10	76	3
	11	23	96	26	10	11	10	68	19	52	88	1	100	33	19	8	46	1	10	16	48	100	100	37	7	100	6
pumA	Time (h)	P1	P2	P3	P4	P5	P6	P7	P8	P9	P10	P11	P12	P13	P14	P15	P16	P17	P18	P19	P20	P21	P22	P23	P24	P25	P26
	0	100	8	100	100	100	84	100	100	100	96	100	100	100	100	100	100	100	100	100	100	100	24	100	25	20	99
	2	85	12	77	24	24	100	84	24	66	61	87	18	38	89	41	85	74	95	11	88	94	93	93	24	34	100
	6	32	36	45	26	30	45	90	44	86	43	57	44	14	29	19	87	36	22	62	55	90	100	45	28	48	34
	8	29	69	20	30	10	19	15	26	95	75	42	26	19	26	33	38	22	35	45	53	57	70	33	68	90	61
	11	19	100	45	2	17	36	13	11	28	100	26	57	28	8	49	3	9	14	65	19	84	25	50	100	100	42
anxA	Time (h)	P1	P2	P3	P4	P5	P6	P7	P8	P9	P10	P11	P12	P13	P14	P15	P16	P17	P18	P19	P20	P21	P22	P23	P24	P25	P26
	0	100	9	100	100	100	71	40	100	100	23	100	100	88	100	100	100	100	58	100	100	9	100	100	100	56	100
	2	19	3	86	34	31	100	100	78	86	43	19	69	73	57	39	79	34	100	25	39	35	20	35	89	68	60
	6	4	3	49	36	42	46	78	15	55	53	42	88	15	29	3	59	43	7	19	28	83	9	13	86	100	47
	8	2	51	50	5	5	12	4	3	55	46	33	52	22	5	4	28	12	28	14	16	100	7	3	30	33	33
	11	7	100	51	1	12	5	5	8	25	100	1	53	100	2	5	0	3	10	9	2	39	15	3	26	16	25
ardA	Time (h)	P1	P2	P3	P4	P5	P6	P7	P8	P9	P10	P11	P12	P13	P14	P15	P16	P17	P18	P19	P20	P21	P22	P23	P24	P25	P26
	0	55	84	75	89	75	43	48	87	77	94	79	42	72	100	79	95	91	100	77	44	15	88	100	38	16	24
	2	46	96	100	100	61	87	100	100	50	84	97	66	100	71	78	97	88	79	74	100	27	53	86	25	47	100
	6	63	91	72	98	100	100	65	89	78	100	98	66	78	74	78	100	100	78	91	34	73	93	85	100	100	15
	8	35	100	99	77	93	20	27	100	72	97	70	69	63	47	85	99	93	76	62	84	100	100	48	25	23	75
	11	100	89	76	73	80	21	32	84	100	77	100	100	51	64	100	94	95	71	100	96	27	84	25	19	17	12
meiB	Time (h)	P1	P2	P3	P4	P5	P6	P7	P8	P9	P10	P11	P12	P13	P14	P15	P16	P17	P18	P19	P20	P21	P22	P23	P24	P25	P26
	0	100	18	98	100	100	100	100	100	90	30	100	94	100	100	100	91	80	100	100	100	100	100	100	81	100	100
	2	16	13	47	16	19	43	53	90	74	23	44	24	78	95	70	100	64	43	7	32	26	24	32	81	91	58
	6	12	23	100	14	19	11	54	48	100	37	71	100	16	100	21	47	100	18	8	24	34	33	24	100	69	47
	8	6	100	68	25	12	10	19	9	36	48	63	38	17	17	28	14	27	18	8	37	42	25	13	55	36	46
	11	10	44	55	3	9	14	26	18	97	100	27	42	18	16	30	9	16	6	2	7	37	22	18	28	17	19
hstA	Time (h)	P1	P2	P3	P4	P5	P6	P7	P8	P9	P10	P11	P12	P13	P14	P15	P16	P17	P18	P19	P20	P21	P22	P23	P24	P25	P26
	0	100	10	8	100	100	89	100	42	40	25	26	100	100	100	100	70	71	100	88	100	68	64	100	15	39	100
	2	77	9	20	63	13	100	86	37	53	21	50	47	17	38	0	25	41	82	79	95	67	100	80	86	31	17
	6	47	89	21	27	8	46	36	35	100	49	100	95	22	40	76	100	100	53	35	44	61	87	62	93	39	2
	8	22	83	23	25	3	14	23	22	37	85	91	67	28	27	71	39	79	51	34	57	46	71	52	73	74	12
	11	12	100	100	29	10	12	30	100	82	100	64	90	99	13	3	50	58	41	100	20	100	45	13	100	100	26
pcnA	Time (h)	P1	P2	P3	P4	P5	P6	P7	P8	P9	P10	P11	P12	P13	P14	P15	P16	P17	P18	P19	P20	P21	P22	P23	P24	P25	P26
	0	100	6	13	100	100	35	94	61	73	42	38	20	100	100	100	30	100	35	6	100	67	100	100	100	16	100
	2	36	15	11	22	16	100	100	9	78	32	73	11	79	89	19	10	11	51	20	44	100	29	19	73	28	23
	6	21	16	29	43	28	69	37	15	100	29	100	47	36	53	1	35	20	100	100	13	18	17	25	24	51	5
	8	15	26	13	16	11	26	36	16	21	49	33	48	28	46	1	21	6	57	75	41	44	13	11	43	100	8
	11	15	100	100	5	15	77	41	100	86	100	34	100	63	28	10	100	1	36	14	58	77	23	10	24	88	14
uchA	Time (h)	P1	P2	P3	P4	P5	P6	P7	P8	P9	P10	P11	P12	P13	P14	P15	P16	P17	P18	P19	P20	P21	P22	P23	P24	P25	P26
	0	94	91	76	100	100	98	90	100	59	73	35	54	100	100	100	100	100	100	82	100	44	100	100	17	30	74
	2	88	82	50	51	61	100	82	21	100	71	64	12	46	61	7	80	83	75	100	64	64	53	37	66	46	72
	6	87	80	55	67	79	41	100	18	91	53	100	100	34	32	3	42	71	70	2	67	94	47	31	100	97	100
	8	100	100	72	70	24	15	10	20	49	100	45	27	23	10	7	20	20	75	6	13	100	40	13	80	100	49
	11	85	90	100	59	42	10	10	9	63	50	43	21	25	3	5	5	10	77	4	7	38	31	17	50	47	36
cudA	Time (h)	P1	P2	P3	P4	P5	P6	P7	P8	P9	P10	P11	P12	P13	P14	P15	P16	P17	P18	P19	P20	P21	P22	P23	P24	P25	P26
	0	100	27	90	100	100	48	20	91	67	31	67	88	100	100	92	83	100	75	37	100	48	100	100	16	65	48
	2	49	8	92	81	63	100	61	100	69	37	69	73	63	80	100	100	63	79	12	28	56	20	29	26	70	62
	6	38	19	93	67	55	58	100	51	65	40	100	100	28	40	32	87	37	100	8	26	56	20	14	58	41	49
	8	20	88	77	7	13	6	7	33	50	93	45	33	21	10	12	92	23	65	17	39	51	15	4	36	100	100
	11	38	100	100	3	11	23	24	29	100	100	41	33	76	11	5	33	10	80	100	10	100	20	6	100	100	56
pikC	Time (h)	P1	P2	P3	P4	P5	P6	P7	P8	P9	P10	P11	P12	P13	P14	P15	P16	P17	P18	P19	P20	P21	P22	P23	P24	P25	P26
	0	100	10	32	44	100	41	18	100	48	67	52	26	79	100	100	74	60	18	100	72	45	100	100	18	37	59
	2	28	38	56	53	68	100	28	8	59	50	81	30	100	92	68	67	100	15	89	49	39	88	50	70	43	85
	6	23	39	79	55	27	77	100	19	100	79	100	100	61	57	84	78	55	100	86	29	62	69	49	97	77	100
	8	15	30	65	63	28	27	26	15	60	75	26	27	43	70	99	100	64	83	98	100	100	76	63	100	100	60
	11	8	100	100	100	27	51	26	41	60	100	25	60	59	71	92	87	68	37	93	31	48	50	55	44	15	61
ribA	Time (h)	P1	P2	P3	P4	P5	P6	P7	P8	P9	P10	P11	P12	P13	P14	P15	P16	P17	P18	P19	P20	P21	P22	P23	P24	P25	P26
	0	100	11	31	100	100	96	80	100	100	71	59	100	100	100	100	100	100	100	100	100	55	64	100	6	11	100
	2	21	17	10	65	53	100	100	40	74	45	79	36	58	40	90	49	55	52	81	71	96	37	22	100	46	100
	6	16	60	31	73	80	49	30	37	77	76	100	42	66	22	67	44	28	42	51	42	100	100	28	75	68	59
	8	7	75	40	69	31	14	30	22	38	93	64	47	41	13	71	38	17	41	38	33	63	82	30	82	90	35
	11	12	100	100	9	34	14	55	39	47	100	45	76	31	14	44	19	18	47	48	35	71	72	13	86	100	17
pksA	Time (h)	P1	P2	P3	P4	P5	P6	P7	P8	P9	P10	P11	P12	P13	P14	P15	P16	P17	P18	P19	P20	P21	P22	P23	P24	P25	P26
	0	69	24	76	100	100	33	56	99	100	42	84	100	100	76	36	62	86	98	19	100	47	26	78	66	15	65
	2	64	64	81	77	35	100	63	100	63	39	56	31	60	82	100	76	49	60	25	31	49	31	20	63	57	53
	6	48	100	100	72	75	80	100	64	92	60	100	61	92	91	82	100	100	75	43	37	97	85	100	15	55	100
	8	30	75	94	77	37	18	58	56	32	72	45	42	37	57	92	77	43	82	63	14	100	81	64	100	68	51
	11	100	36	87	96	32	48	64	63	48	100	33	60	46	100	65	78	43	100	100	22	52	100	58	76	100	25
dspA	Time (h)	P1	P2	P3	P4	P5	P6	P7	P8	P9	P10	P11	P12	P13	P14	P15	P16	P17	P18	P19	P20	P21	P22	P23	P24	P25	P26
	0	91	86	90	48	88	97	89	100	82	96	94	95	90	99	94	94	0	0	92	100	91	93	90	0	0	74
	2	92	78	100	43	100	94	92	95	100	99	95	100	100	99	0	94	96	99	97	87	88	93	91	87	94	100
	6	99	91	92	100	99	93	92	94	94	100	96	100	58	100	0	100	100	98	100	89	87	97	100	92	93	89
	8	100	95	91	46	95	100	100	92	90	93	100	95	93	0	100	95	95	98	95	89	100	90	94	100	100	82
	11	98	100	97	49	91	91	90	96	86	92	90	93	91	99	98	0	0	100	96	100	89	100	89	100	99	71
ribB	Time (h)	P1	P2	P3	P4	P5	P6	P7	P8	P9	P10	P11	P12	P13	P14	P15	P16	P17	P18	P19	P20	P21	P22	P23	P24	P25	P26
	0	95	100	96	95	89	78	62	99	100	100	97	87	92	95	98	93	100	100	95	93	71	100	86	87	91	93
	2	95	85	95	95	85	100	100	100	89	98	98	100	83	89	99	79	65	72	93	99	100	91	67	83	100	89
	6	90	85	100	100	100	77	80	86	89	97	100	91	95	100	92	72	95	79	100	100	84	92	97	83	93	94
	8	100	98	97	92	94	81	73	86	88	96	96	86	100	83	90	77	71	79	83	97	73	94	100	100	91	95
	11	96	98	97	99	97	82	79	93	87	96	98	81	99	97	100	100	92	86	98	91	84	91	93	96	89	100
hcpA	Time (h)	P1	P2	P3	P4	P5	P6	P7	P8	P9	P10	P11	P12	P13	P14	P15	P16	P17	P18	P19	P20	P21	P22	P23	P24	P25	P26
	0	100	8	4	100	100	3	6	16	8	2	15	2	11	15	1	14	100	44	12	100	51	100	100	17	44	16
	2	51	22	7	2	5	65	22	5	13	9	10	45	15	9	2	5	20	97	31	57	31	40	42	100	96	58
	6	49	18	27	8	16	100	36	75	33	20	52	46	24	33	8	38	8	70	62	49	34	16	20	78	41	32
	8	30	68	38	1	4	66	44	79	71	100	43	49	39	74	34	91	8	97	100	65	38	13	6	100	56	78
	11	89	100	100	3	8	66	100	100	100	95	100	100	100	100	100	100	5	100	79	30	100	25	11	77	100	100
damA	Time (h)	P1	P2	P3	P4	P5	P6	P7	P8	P9	P10	P11	P12	P13	P14	P15	P16	P17	P18	P19	P20	P21	P22	P23	P24	P25	P26
	0	44	9	81	100	100	100	25	71	88	20	30	15	100	74	25	100	100	74	100	46	57	41	22	21	87	100
	2	100	13	34	22	46	76	93	45	58	22	36	51	73	96	93	98	44	100	31	100	75	33	30	100	81	98
	6	67	56	100	97	80	17	100	58	100	36	100	82	54	90	17	97	58	64	36	75	90	52	100	28	76	100
	8	59	76	25	55	98	20	39	48	63	88	92	89	48	79	100	97	36	52	12	65	100	42	41	22	100	91
	11	80	100	71	27	64	30	85	100	96	100	73	100	56	100	55	95	20	63	74	77	98	100	44	49	51	98
arpA	Time (h)	P1	P2	P3	P4	P5	P6	P7	P8	P9	P10	P11	P12	P13	P14	P15	P16	P17	P18	P19	P20	P21	P22	P23	P24	P25	P26
	0	19	11	24	100	35	5	8	89	31	34	16	40	90	26	40	20	83	15	18	20	33	8	14	17	50	19
	2	48	56	15	15	13	9	17	100	29	39	15	5	49	24	74	33	24	18	19	23	65	51	52	18	58	36
	6	73	94	47	21	100	24	16	61	100	100	61	100	47	31	100	72	100	79	16	100	100	82	51	39	58	100
	8	92	66	77	37	38	51	36	50	45	71	60	35	100	57	77	100	65	87	80	26	88	61	68	52	90	47
	11	100	100	100	17	30	100	100	78	36	82	100	18	40	100	28	100	71	100	100	45	93	100	100	100	100	74
tspA	Time (h)	P1	P2	P3	P4	P5	P6	P7	P8	P9	P10	P11	P12	P13	P14	P15	P16	P17	P18	P19	P20	P21	P22	P23	P24	P25	P26
	0	5	5	32	100	52	18	96	67	55	56	92	43	58	72	26	80	44	25	57	100	59	40	100	58	29	43
	2	13	18	57	19	44	62	100	29	52	39	78	15	82	38	100	30	34	100	34	21	63	100	32	64	47	100
	6	11	17	83	44	100	56	93	24	100	55	83	100	93	100	15	100	100	97	44	22	32	62	59	55	63	49
	8	10	25	70	15	47	29	56	42	34	100	91	29	63	37	26	47	21	77	34	73	100	48	43	100	100	36
	11	100	100	100	10	81	100	83	100	92	56	100	17	100	43	62	99	40	32	100	52	94	53	25	57	32	47
rasA	Time (h)	P1	P2	P3	P4	P5	P6	P7	P8	P9	P10	P11	P12	P13	P14	P15	P16	P17	P18	P19	P20	P21	P22	P23	P24	P25	P26
	0	20	3	7	31	75	9	13	43	32	10	62	75	44	13	24	16	14	9	14	13	5	31	100	1	15	21
	2	24	1	13	43	42	83	31	34	22	11	61	81	59	18	78	33	43	51	41	36	8	44	20	32	66	35
	6	30	8	19	45	100	100	100	33	54	28	90	100	63	23	84	100	48	66	80	24	26	48	22	79	79	100
	8	50	19	22	54	35	71	62	40	78	46	100	93	100	27	100	78	54	100	100	21	26	78	25	100	95	72
	11	100	100	100	100	69	79	64	100	100	100	88	62	46	100	94	66	100	83	89	100	100	100	66	91	100	95
pptA	Time (h)	P1	P2	P3	P4	P5	P6	P7	P8	P9	P10	P11	P12	P13	P14	P15	P16	P17	P18	P19	P20	P21	P22	P23	P24	P25	P26
	0	9	5	8	9	2	0	3	3	3	2	2	31	1	3	2	4	7	3	33	24	50	59	6	0	21	6
	2	1	8	45	1	1	6	6	0	3	2	6	1	2	9	44	65	17	12	62	37	100	59	3	20	60	67
	6	9	19	50	100	19	89	47	8	100	47	75	100	100	40	60	48	83	22	34	29	29	18	29	11	69	50
	8	13	63	100	66	16	100	35	16	52	100	100	48	54	61	85	62	64	87	25	73	66	48	17	81	39	25
	11	100	100	77	86	100	71	100	100	63	77	59	46	22	100	100	100	100	100	100	100	17	100	100	100	100	100
ehdA	Time (h)	P1	P2	P3	P4	P5	P6	P7	P8	P9	P10	P11	P12	P13	P14	P15	P16	P17	P18	P19	P20	P21	P22	P23	P24	P25	P26
	0	6	7	12	73	74	4	9	31	28	8	14	24	74	11	6	6	19	29	100	10	9	16	90	7	10	25
	2	11	19	22	18	37	54	44	31	19	10	16	27	40	14	14	59	25	65	62	10	14	23	19	40	100	40
	6	11	51	32	100	94	79	100	32	87	25	70	52	80	39	24	57	26	48	67	16	23	27	100	100	53	0
	8	33	74	52	23	39	94	46	25	80	75	79	44	100	40	51	51	31	100	67	21	50	40	49	25	50	56
	11	100	100	100	37	100	100	92	100	100	100	100	100	52	100	100	100	100	97	90	100	100	100	40	37	78	100
spiA	Time (h)	P1	P2	P3	P4	P5	P6	P7	P8	P9	P10	P11	P12	P13	P14	P15	P16	P17	P18	P19	P20	P21	P22	P23	P24	P25	P26
	0	40	10	13	16	20	7	2	18	17	4	43	41	33	17	6	18	8	1	34	100	5	64	100	1	30	7
	2	35	4	54	1	6	37	10	6	17	9	11	4	32	21	0	91	15	18	21	17	6	63	12	16	47	100
	6	42	20	37	100	86	100	100	32	52	25	95	100	100	21	11	90	100	100	58	33	20	51	42	33	55	52
	8	50	44	37	0	100	90	84	13	49	100	66	31	62	25	51	64	37	44	89	52	35	37	19	100	59	25
	11	100	100	100	13	71	86	63	100	100	95	100	37	89	100	100	100	9	100	100	100	100	100	11	69	100	37
cdcA	Time (h)	P1	P2	P3	P4	P5	P6	P7	P8	P9	P10	P11	P12	P13	P14	P15	P16	P17	P18	P19	P20	P21	P22	P23	P24	P25	P26
	0	17	4	14	37	12	15	9	24	29	13	16	16	16	26	3	8	7	12	32	45	83	12	15	1	14	29
	2	8	9	50	24	8	100	44	23	31	12	26	20	12	21	100	40	7	26	59	26	78	51	47	8	17	72
	6	19	90	49	43	62	95	90	29	34	36	100	40	86	99	28	50	50	66	92	42	80	57	96	18	58	100
	8	14	82	38	100	61	48	88	26	57	78	63	83	83	83	72	99	50	100	66	70	100	58	88	89	31	89
	11	100	100	100	87	100	42	100	100	100	100	51	100	100	100	34	100	100	95	100	100	54	100	100	100	100	87
pakA	Time (h)	P1	P2	P3	P4	P5	P6	P7	P8	P9	P10	P11	P12	P13	P14	P15	P16	P17	P18	P19	P20	P21	P22	P23	P24	P25	P26
	0	8	24	20	10	10	13	11	4	29	15	23	7	15	11	4	11	7	11	8	15	61	6	9	2	6	33
	2	19	76	39	39	9	73	38	55	35	10	42	12	13	18	100	100	6	14	12	21	71	36	14	3	21	49
	6	18	71	55	32	73	72	42	36	100	36	95	100	100	40	80	82	100	73	34	24	60	94	100	21	50	50
	8	16	63	53	100	87	100	60	39	40	77	91	96	88	100	90	51	71	87	58	19	78	79	63	19	55	52
	11	100	100	100	78	100	77	100	100	0	100	100	68	98	94	81	59	92	100	100	100	100	100	63	100	100	100
gapA	Time (h)	P1	P2	P3	P4	P5	P6	P7	P8	P9	P10	P11	P12	P13	P14	P15	P16	P17	P18	P19	P20	P21	P22	P23	P24	P25	P26
	0	9	2	5	27	24	3	6	10	11	4	8	33	15	5	5	2	26	18	37	38	8	36	60	0	18	8
	2	5	1	5	28	14	27	24	2	11	10	42	27	15	52	16	18	28	40	38	26	14	81	77	24	19	13
	6	15	15	6	80	99	28	17	36	37	28	47	73	100	53	30	29	52	63	65	12	27	69	80	70	69	18
	8	19	48	20	62	75	59	19	7	81	70	81	100	97	58	67	97	59	100	49	32	40	92	89	100	70	80
	11	100	100	100	100	100	100	100	100	100	100	100	99	55	100	100	100	100	65	100	100	100	100	100	97	100	100
pldA	Time (h)	P1	P2	P3	P4	P5	P6	P7	P8	P9	P10	P11	P12	P13	P14	P15	P16	P17	P18	P19	P20	P21	P22	P23	P24	P25	P26
	0	10	5	9	3	1	4	4	5	9	2	2	14	26	1	0	2	4	0	5	7	23	1	14	24	4	17
	2	31	3	96	2	3	100	96	3	21	11	12	4	19	4	17	19	15	7	7	16	49	36	27	5	15	33
	6	51	93	74	34	100	36	59	100	22	25	82	37	67	15	38	75	100	38	39	15	63	45	100	100	18	44
	8	49	100	48	100	70	23	100	36	73	46	60	28	100	33	58	97	67	100	38	13	100	90	89	99	79	65
	11	100	85	100	78	99	15	66	37	100	100	100	100	94	100	100	100	51	76	100	100	72	100	94	78	100	100
ligA	Time (h)	P1	P2	P3	P4	P5	P6	P7	P8	P9	P10	P11	P12	P13	P14	P15	P16	P17	P18	P19	P20	P21	P22	P23	P24	P25	P26
	0	5	3	8	27	34	3	3	16	8	8	6	2	15	4	2	1	28	27	7	8	12	3	10	1	1	4
	2	5	12	10	97	26	18	9	10	10	4	6	12	25	7	7	29	11	20	16	14	17	51	61	14	2	16
	6	18	67	9	100	30	41	64	18	43	23	40	34	43	100	23	55	100	28	82	13	16	43	100	71	5	11
	8	34	100	51	100	21	20	76	31	54	81	80	53	62	94	92	68	60	24	99	40	45	55	85	87	9	27
	11	100	93	100	94	100	100	100	100	100	100	100	100	100	86	100	100	63	100	100	100	100	100	73	100	100	100
pwiA	Time (h)	P1	P2	P3	P4	P5	P6	P7	P8	P9	P10	P11	P12	P13	P14	P15	P16	P17	P18	P19	P20	P21	P22	P23	P24	P25	P26
	0	6	2	27	7	3	0	2	5	1	1	5	5	1	0	4	1	7	4	5	5	13	3	19	0	1	2
	2	1	1	12	1	2	9	3	0	3	1	5	1	1	2	22	50	4	49	11	3	19	6	4	24	6	9
	6	6	21	29	91	15	33	37	10	23	14	47	28	31	26	16	83	44	72	35	1	20	11	34	51	8	16
	8	13	82	85	77	23	21	23	15	52	96	100	41	61	36	100	100	73	100	64	1	85	7	48	72	30	41
	11	100	100	100	100	100	100	100	100	100	100	45	100	100	100	100	90	100	47	100	100	100	100	100	100	100	100
rgsA	Time (h)	P1	P2	P3	P4	P5	P6	P7	P8	P9	P10	P11	P12	P13	P14	P15	P16	P17	P18	P19	P20	P21	P22	P23	P24	P25	P26
	0	1	44	7	2	4	1	0	6	1	1	1	3	1	2	1	1	6	0	32	3	23	2	8	0	2	4
	2	1	0	47	0	2	7	3	0	2	1	1	0	2	6	11	7	23	17	22	5	35	7	16	3	18	10
	6	2	100	13	52	15	18	12	2	4	7	5	31	29	22	13	9	100	27	0	3	24	26	57	23	13	43
	8	3	30	14	1	15	22	18	3	13	34	14	39	26	50	19	20	57	100	37	0	69	19	100	89	37	75
	11	100	22	100	100	100	100	100	100	100	100	100	100	100	100	100	100	25	71	100	100	100	100	97	100	100	100
		6d, 5 min FR				6d, 15 min FR									7d, 20 min FR				7d, 30 min FR								

		6d, 5 min FR				6d, 15 min FR									7d, 20 min FR				7d, 30 min FR								
	Time (h)	P1	P2	P3	P4	P5	P6	P7	P8	P9	P10	P11	P12	P13	P14	P15	P16	P17	P18	P19	P20	P21	P22	P23	P24	P25	P26
psgA	0	100	100	100	100	100	100	89	100	100	100	100	100	100	100	100	100	100	100	100	100	100	100	100	100	100	76
	2	59	47	68	71	34	25	48	35	98	83	57	78	74	45	25	3	51	28	8	27	71	11	20	24	88	100
	6	7	29	52	28	17	1	100	21	57	20	19	72	12	25	1	27	16	32	2	17	54	10	4	11	44	4
	8	3	5	18	24	2	1	10	15	12	16	14	17	6	3	1	8	3	7	0	16	46	13	3	7	46	12
	11	13	12	9	1	0	0	2	3	8	7	2	21	10	0	0	1	1	1	0	0	0	0	0	0	0	6
pldC	Time (h)	P1	P2	P3	P4	P5	P6	P7	P8	P9	P10	P11	P12	P13	P14	P15	P16	P17	P18	P19	P20	P21	P22	P23	P24	P25	P26
	0	100	38	100	100	33	100	100	100	58	100	100	22	25	100	100	100	100	100	100	100	61	100	100	100	83	100
	2	12	61	82	27	100	7	42	2	47	21	40	100	31	35	100	2	24	80	32	15	43	4	45	15	1	20
	6	2	28	2	15	13	7	8	3	28	2	6	48	28	18	7	4	30	41	30	11	7	2	5	1	34	2
	8	1	100	0	5	17	6	1	1	37	3	1	7	26	16	4	3	9	69	1	11	100	2	2	1	100	1
	11	1	49	2	2	10	1	1	1	100	8	1	11	100	5	3	1	4	27	1	1	39	5	1	0	1	6
pptB	Time (h)	P1	P2	P3	P4	P5	P6	P7	P8	P9	P10	P11	P12	P13	P14	P15	P16	P17	P18	P19	P20	P21	P22	P23	P24	P25	P26
	0	100	11	19	81	100	100	100	100	61	46	64	23	100	100	100	100	100	100	67	100	35	100	100	16	27	100
	2	22	49	59	31	51	48	0	89	38	38	88	12	41	58	51	73	66	45	21	84	29	85	43	66	56	82
	6	20	57	40	100	50	10	21	36	100	33	100	89	22	37	10	59	32	87	40	51	100	91	75	100	70	1
	8	16	60	21	60	36	19	28	74	21	51	93	81	11	24	11	24	25	73	76	88	40	81	43	80	100	86
	11	5	100	100	16	44	9	35	70	41	100	79	100	15	16	3	10	5	25	100	37	32	21	24	57	17	47
pldB	Time (h)	P1	P2	P3	P4	P5	P6	P7	P8	P9	P10	P11	P12	P13	P14	P15	P16	P17	P18	P19	P20	P21	P22	P23	P24	P25	P26
	0	100	0	100	0	46	100	0	0	0	100	0	26	0	100	100	98	0	100	100	100	24	65	69	38	31	51
	2	16	1	38	9	41	4	0	100	100	79	100	17	100	36	16	100	38	90	51	55	50	76	100	59	20	74
	6	12	100	74	100	100	30	100	44	46	76	80	100	0	30	30	14	100	64	34	46	100	100	41	100	100	100
	8	0	98	3	22	0	0	5	4	25	50	0	24	0	0	0	13	6	52	27	11	0	0	34	70	46	25
	11	2	0	3	0	0	0	3	0	0	0	7	0	0	0	0	2	13	48	0	24	0	0	0	0	19	0
pikB	Time (h)	P1	P2	P3	P4	P5	P6	P7	P8	P9	P10	P11	P12	P13	P14	P15	P16	P17	P18	P19	P20	P21	P22	P23	P24	P25	P26
	0	100	37	100	100	100	100	100	100	100	70	100	76	100	100	100	100	100	100	100	100	73	100	100	100	100	100
	2	32	11	27	6	7	21	0	13	92	40	25	58	81	52	51	34	34	26	40	99	100	96	22	98	56	39
	6	18	66	39	11	15	21	83	35	77	18	68	100	43	25	21	34	12	6	21	100	14	37	13	71	62	19
	8	16	76	20	8	13	4	0	7	53	100	35	43	53	21	4	25	5	8	14	69	12	45	4	77	42	37
	11	10	100	17	4	17	27	26	11	51	77	15	34	80	17	3	14	4	9	2	43	16	0	3	68	30	35
ralA	Time (h)	P1	P2	P3	P4	P5	P6	P7	P8	P9	P10	P11	P12	P13	P14	P15	P16	P17	P18	P19	P20	P21	P22	P23	P24	P25	P26
	0	100	18	100	100	93	100	100	100	100	100	100	100	100	100	100	78	100	100	100	100	100	100	100	4	100	100
	2	25	57	94	57	100	16	0	21	92	76	85	65	90	80	41	88	81	25	50	17	74	59	21	33	68	15
	6	9	45	61	68	58	28	21	18	46	58	74	16	55	71	28	100	8	29	42	5	52	38	7	59	51	16
	8	7	100	54	11	17	6	17	18	16	40	0	37	15	33	11	52	6	27	9	12	94	37	11	100	25	37
	11	5	78	52	5	16	5	35	17	4	38	18	29	7	8	4	10	1	11	6	5	71	71	12	75	2	35
nhpA	Time (h)	P1	P2	P3	P4	P5	P6	P7	P8	P9	P10	P11	P12	P13	P14	P15	P16	P17	P18	P19	P20	P21	P22	P23	P24	P25	P26
	0	100	27	100	100	91	100	14	100	100	100	100	47	77	100	100	64	100	100	69	100	7	10	100	100	1	100
	2	33	52	84	37	100	27	100	28	72	57	56	55	100	30	7	56	60	2	100	21	100	59	41	53	8	51
	6	5	100	15	63	54	2	83	22	44	39	39	0	22	21	2	100	15	20	36	36	4	22	77	37	27	2
	8	10	99	12	51	7	2	16	9	10	60	22	13	17	16	3	25	1	17	46	44	5	18	34	8	77	3
	11	22	100	25	17	15	5	72	17	39	91	1	100	26	10	5	50	2	21	10	48	7	100	29	5	100	6
pumA	Time (h)	P1	P2	P3	P4	P5	P6	P7	P8	P9	P10	P11	P12	P13	P14	P15	P16	P17	P18	P19	P20	P21	P22	P23	P24	P25	P26
	0	100	9	100	100	100	100	94	100	100	100	100	100	100	100	100	100	100	100	100	100	81	23	100	17	19	85
	2	86	13	69	23	28	33	0	29	70	80	91	16	39	82	55	76	85	100	18	73	0	100	99	19	28	100
	6	33	41	49	27	36	5	70	51	87	34	69	40	14	52	5	91	48	21	51	62	96	97	25	20	47	38
	8	30	71	14	27	10	6	100	22	99	75	43	25	18	40	13	36	16	30	29	46	60	67	32	66	86	68
	11	25	100	47	6	21	23	36	14	46	91	38	62	28	18	36	4	8	11	56	14	100	4	49	100	100	43
anxA	Time (h)	P1	P2	P3	P4	P5	P6	P7	P8	P9	P10	P11	P12	P13	P14	P15	P16	P17	P18	P19	P20	P21	P22	P23	P24	P25	P26
	0	100	11	100	100	100	100	32	100	100	35	100	100	90	100	100	100	100	88	100	100	21	100	100	100	74	100
	2	19	5	80	35	25	10	100	64	71	48	19	74	75	55	55	84	32	100	31	39	50	28	36	85	100	69
	6	4	4	39	38	46	1	68	12	58	62	28	85	15	39	1	60	51	7	12	28	70	9	22	77	86	65
	8	2	51	41	5	10	2	7	3	51	52	22	38	25	13	1	29	11	38	6	11	100	8	8	35	39	51
	11	5	100	41	0	13	1	8	6	30	100	12	49	100	5	0	6	4	10	5	1	51	19	4	27	12	7
ardA	Time (h)	P1	P2	P3	P4	P5	P6	P7	P8	P9	P10	P11	P12	P13	P14	P15	P16	P17	P18	P19	P20	P21	P22	P23	P24	P25	P26
	0	52	83	70	93	78	100	75	62	59	100	83	25	78	100	59	100	94	100	91	65	15	74	100	61	12	40
	2	53	90	100	100	76	34	0	100	41	90	97	43	100	60	61	100	78	62	98	88	6	50	89	19	47	100
	6	78	92	57	81	100	93	100	68	49	92	98	54	87	57	55	99	95	70	100	30	72	72	90	100	100	11
	8	30	100	91	67	91	20	52	57	85	80	70	46	66	54	61	99	96	69	84	79	100	100	30	29	33	79
	11	100	80	71	66	78	27	38	29	100	63	100	100	54	33	100	98	100	67	94	100	29	78	19	35	13	12
meiB	Time (h)	P1	P2	P3	P4	P5	P6	P7	P8	P9	P10	P11	P12	P13	P14	P15	P16	P17	P18	P19	P20	P21	P22	P23	P24	P25	P26
	0	100	18	100	100	100	100	100	100	76	35	100	100	100	100	100	87	68	100	100	100	100	100	100	94	93	100
	2	23	10	54	19	22	95	60	91	73	19	49	23	83	96	76	100	68	47	8	38	38	21	22	91	100	53
	6	16	18	95	20	15	24	57	49	100	27	58	97	15	75	24	61	100	12	8	27	29	36	10	100	75	51
	8	7	100	66	29	13	7	13	9	47	47	49	44	21	18	31	27	11	10	7	36	49	20	7	61	34	40
	11	9	51	94	5	10	13	24	12	97	100	43	50	22	17	22	10	6	4	0	7	32	18	24	27	17	7
hstA	Time (h)	P1	P2	P3	P4	P5	P6	P7	P8	P9	P10	P11	P12	P13	P14	P15	P16	P17	P18	P19	P20	P21	P22	P23	P24	P25	P26
	0	100	10	8	100	100	94	100	37	43	21	19	100	91	100	100	59	78	100	89	100	82	63	100	15	27	100
	2	85	8	23	79	13	31	60	27	51	18	49	42	17	38	3	35	60	77	55	88	21	100	80	89	17	18
	6	46	90	26	51	8	84	32	22	100	48	100	97	22	46	89	100	100	58	47	47	55	80	70	100	48	6
	8	25	83	30	15	3	100	22	26	30	93	66	77	51	34	94	38	81	59	51	26	48	72	53	77	69	14
	11	13	100	100	53	3	39	31	100	88	100	67	83	100	6	3	38	59	41	100	17	100	42	11	99	100	26
pcnA	Time (h)	P1	P2	P3	P4	P5	P6	P7	P8	P9	P10	P11	P12	P13	P14	P15	P16	P17	P18	P19	P20	P21	P22	P23	P24	P25	P26
	0	100	5	11	100	100	100	100	71	49	43	42	21	100	100	100	40	100	23	8	100	41	100	100	100	15	100
	2	38	14	9	24	17	38	100	12	71	30	71	17	71	63	13	18	16	52	32	78	100	34	16	54	20	26
	6	24	16	25	37	25	1	55	17	100	26	100	40	39	55	1	71	23	100	100	29	11	25	41	18	48	15
	8	14	26	12	22	11	56	25	18	28	59	0	44	29	51	1	29	6	67	73	33	31	25	8	29	100	18
	11	14	100	100	7	18	24	33	100	91	100	37	100	68	6	9	100	1	25	17	98	49	14	4	17	65	19
uchA	Time (h)	P1	P2	P3	P4	P5	P6	P7	P8	P9	P10	P11	P12	P13	P14	P15	P16	P17	P18	P19	P20	P21	P22	P23	P24	P25	P26
	0	97	63	68	100	100	100	94	100	70	85	32	48	100	100	100	100	100	100	90	100	29	100	100	34	28	83
	2	86	59	69	50	46	15	72	5	98	65	63	10	44	59	11	72	83	71	100	69	23	57	25	79	34	55
	6	93	59	78	72	88	1	100	23	100	55	100	100	31	37	1	42	53	61	1	72	87	58	35	100	97	100
	8	100	100	78	82	25	35	9	25	58	100	0	23	23	3	5	16	21	55	8	16	100	50	12	76	100	58
	11	89	89	100	51	40	9	14	6	60	53	52	19	22	2	7	6	14	59	4	15	16	37	16	43	32	33
cudA	Time (h)	P1	P2	P3	P4	P5	P6	P7	P8	P9	P10	P11	P12	P13	P14	P15	P16	P17	P18	P19	P20	P21	P22	P23	P24	P25	P26
	0	100	27	71	100	100	100	26	100	64	30	63	87	100	100	84	81	100	76	39	100	27	100	100	20	68	47
	2	56	16	78	86	78	18	45	99	55	35	63	85	60	80	100	100	54	87	12	33	9	15	25	29	75	65
	6	36	17	73	60	33	34	100	87	47	47	100	100	33	44	29	87	36	100	3	23	41	16	18	72	45	49
	8	27	89	37	7	17	2	3	76	38	77	49	25	12	5	11	18	19	63	26	44	35	14	6	68	95	100
	11	33	100	100	3	16	7	11	16	100	100	49	25	66	16	5	2	9	59	100	8	100	17	8	100	100	75
pikC	Time (h)	P1	P2	P3	P4	P5	P6	P7	P8	P9	P10	P11	P12	P13	P14	P15	P16	P17	P18	P19	P20	P21	P22	P23	P24	P25	P26
	0	100	11	27	30	100	66	12	100	54	34	56	30	64	100	66	61	67	22	100	69	40	100	100	21	32	48
	2	33	48	45	64	52	91	21	10	52	26	88	33	100	82	53	59	100	21	98	57	100	70	27	24	41	60
	6	21	46	100	62	26	100	100	12	100	87	100	100	68	59	100	74	60	100	95	22	38	43	38	98	85	100
	8	15	25	59	51	24	16	42	12	69	39	42	52	32	66	86	100	74	99	96	100	59	42	97	100	100	43
	11	9	100	90	100	23	35	55	31	76	100	24	60	46	72	78	79	68	60	98	29	28	21	79	27	16	31
ribA	Time (h)	P1	P2	P3	P4	P5	P6	P7	P8	P9	P10	P11	P12	P13	P14	P15	P16	P17	P18	P19	P20	P21	P22	P23	P24	P25	P26
	0	100	9	34	100	100	81	78	100	100	60	62	100	100	100	100	100	100	100	100	100	30	39	100	5	10	88
	2	23	15	11	60	44	100	100	44	73	50	89	35	54	29	94	60	56	47	74	74	100	13	24	100	44	100
	6	16	35	34	66	91	56	27	29	78	80	100	0	65	18	69	48	17	45	47	47	69	100	42	91	76	54
	8	9	67	39	64	25	15	35	20	47	94	62	58	43	11	83	37	14	40	32	45	38	95	29	97	93	27
	11	10	100	100	7	37	18	62	34	58	100	51	76	30	10	49	17	13	55	52	45	45	59	13	94	100	15
pksA	Time (h)	P1	P2	P3	P4	P5	P6	P7	P8	P9	P10	P11	P12	P13	P14	P15	P16	P17	P18	P19	P20	P21	P22	P23	P24	P25	P26
	0	72	27	68	100	100	15	56	97	100	49	90	100	100	63	23	63	82	100	19	100	34	25	64	67	10	68
	2	61	70	94	73	54	100	70	100	77	47	59	30	59	94	100	75	53	64	32	26	100	25	22	67	43	54
	6	47	100	100	69	66	48	100	59	85	65	100	58	83	100	75	100	100	83	50	30	62	80	100	19	43	100
	8	37	84	88	78	42	17	62	44	33	84	41	38	33	52	87	68	49	94	62	16	81	65	61	100	68	50
	11	100	41	82	100	43	39	66	64	33	100	36	63	40	85	51	75	25	98	100	15	40	100	49	71	100	20
dspA	Time (h)	P1	P2	P3	P4	P5	P6	P7	P8	P9	P10	P11	P12	P13	P14	P15	P16	P17	P18	P19	P20	P21	P22	P23	P24	P25	P26
	0	98	98	85	63	64	99	86	100	95	95	95	92	87	98	98	85	95	98	99	100	73	64	100	98	71	100
	2	83	92	100	63	0	82	0	84	100	98	89	100	100	98	99	88	99	96	98	97	46	70	100	98	80	99
	6	98	92	99	96	77	100	94	80	97	87	100	94	52	100	99	100	100	87	98	100	78	100	96	98	76	84
	8	99	100	87	84	100	75	100	91	86	97	0	97	89	99	100	83	99	100	100	96	100	64	97	100	98	94
	11	100	98	86	100	0	77	98	83	97	100	81	100	82	98	94	85	97	100	99	98	74	100	98	98	100	84
ribB	Time (h)	P1	P2	P3	P4	P5	P6	P7	P8	P9	P10	P11	P12	P13	P14	P15	P16	P17	P18	P19	P20	P21	P22	P23	P24	P25	P26
	0	92	100	100	89	96	98	72	91	100	98	100	90	94	99	98	100	86	100	98	96	95	99	100	99	85	93
	2	94	88	92	96	95	63	100	100	88	99	92	97	80	90	99	75	72	86	99	99	85	99	57	100	100	97
	6	90	84	98	92	100	100	88	82	86	100	95	100	90	100	100	66	97	87	99	100	100	100	64	99	95	100
	8	100	99	88	90	98	52	83	79	91	100	0	89	100	97	92	70	92	75	100	98	96	98	93	95	100	93
	11	94	92	87	100	96	56	89	88	87	99	92	92	100	96	94	98	100	71	98	97	95	100	75	95	98	96
hcpA	Time (h)	P1	P2	P3	P4	P5	P6	P7	P8	P9	P10	P11	P12	P13	P14	P15	P16	P17	P18	P19	P20	P21	P22	P23	P24	P25	P26
	0	97	15	5	4	100	17	8	37	9	2	12	1	10	7	2	13	100	54	11	100	45	100	100	8	42	9
	2	29	21	8	100	6	94	35	5	14	10	7	29	14	11	3	7	20	99	26	64	13	47	22	53	100	52
	6	31	20	28	6	21	41	36	78	33	23	54	38	24	20	5	31	6	69	65	59	31	15	19	39	29	25
	8	19	72	37	14	7	81	40	79	63	100	50	41	37	35	41	100	8	83	100	42	37	5	21	52	47	85
	11	100	100	100	9	11	100	100	100	100	96	100	100	100	100	100	100	5	100	71	24	100	4	13	100	97	100
damA	Time (h)	P1	P2	P3	P4	P5	P6	P7	P8	P9	P10	P11	P12	P13	P14	P15	P16	P17	P18	P19	P20	P21	P22	P23	P24	P25	P26
	0	34	9	69	100	100	31	21	65	78	25	33	14	100	83	20	100	100	72	100	56	59	28	26	14	100	93
	2	100	13	21	20	28	72	95	46	51	34	37	48	83	100	100	100	42	100	48	100	28	36	38	100	74	91
	6	57	56	100	98	90	12	100	48	100	36	100	75	50	80	8	100	44	78	21	64	82	47	100	48	61	100
	8	51	71	37	48	100	23	31	49	43	98	94	90	44	70	62	100	19	60	48	60	100	48	44	30	84	80
	11	67	100	56	20	65	100	88	100	79	100	77	100	49	85	44	100	16	78	97	83	84	100	38	54	18	93
arpA	Time (h)	P1	P2	P3	P4	P5	P6	P7	P8	P9	P10	P11	P12	P13	P14	P15	P16	P17	P18	P19	P20	P21	P22	P23	P24	P25	P26
	0	25	11	28	100	35	17	12	96	27	31	15	100	80	21	44	17	75	13	30	24	33	12	26	13	43	28
	2	51	58	19	12	16	6	25	100	27	33	14	22	49	19	83	33	24	17	31	29	21	33	29	24	53	55
	6	72	100	44	13	100	39	20	70	100	47	56	25	48	28	100	73	100	69	37	100	94	86	57	41	49	98
	8	85	76	84	43	48	27	47	53	47	75	58	80	100	50	80	100	39	82	86	37	85	57	70	54	100	70
	11	100	92	100	23	25	100	100	74	25	100	100	40	37	100	29	100	58	100	100	47	100	100	100	100	91	100
tspA	Time (h)	P1	P2	P3	P4	P5	P6	P7	P8	P9	P10	P11	P12	P13	P14	P15	P16	P17	P18	P19	P20	P21	P22	P23	P24	P25	P26
	0	3	4	23	100	64	15	78	50	44	90	93	35	47	97	28	64	63	20	65	100	53	33	100	67	18	52
	2	10	21	64	17	37	49	0	28	36	50	74	14	87	61	100	33	38	100	19	45	100	100	58	48	38	100
	6	10	22	86	30	95	7	100	26	99	63	80	100	100	100	13	100	100	71	30	32	25	81	55	47	62	34
	8	10	27	90	10	52	31	87	38	38	100	84	27	66	67	28	47	22	50	18	87	80	58	46	100	100	35
	11	100	100	100	12	100	100	90	100	100	70	100	13	99	63	63	99	42	27	100	84	78	59	24	58	28	48
rasA	Time (h)	P1	P2	P3	P4	P5	P6	P7	P8	P9	P10	P11	P12	P13	P14	P15	P16	P17	P18	P19	P20	P21	P22	P23	P24	P25	P26
	0	22	3	9	36	84	49	15	40	24	11	47	69	39	11	31	17	0	9	20	21	8	30	100	3	15	24
	2	21	1	12	50	32	36	33	37	23	13	49	73	59	16	100	35	32	41	39	54	36	44	21	41	72	33
	6	34	10	27	53	100	100	100	34	60	29	81	93	60	21	63	100	35	63	81	48	29	46	36	91	85	100
	8	48	18	27	74	47	42	64	49	91	36	0	100	100	24	89	79	55	100	100	33	35	79	61	100	100	71
	11	100	100	100	100	80	45	68	100	100	100	100	84	48	100	89	68	100	81	99	100	100	100	73	100	90	86
pptA	Time (h)	P1	P2	P3	P4	P5	P6	P7	P8	P9	P10	P11	P12	P13	P14	P15	P16	P17	P18	P19	P20	P21	P22	P23	P24	P25	P26
	0	9	5	7	10	4	2	3	5	3	1	3	26	1	2	1	2	7	3	39	20	65	42	8	0	25	6
	2	1	8	42	1	1	2	5	1	3	2	5	1	1	6	27	60	13	11	63	38	100	56	6	24	81	67
	6	12	19	51	100	23	77	48	8	100	41	70	100	100	35	62	44	68	24	30	25	33	24	27	13	100	61
	8	14	64	100	67	42	100	67	14	70	100	100	39	55	60	100	48	55	88	22	71	84	34	10	92	44	43
	11	100	100	61	92	100	80	100	100	55	88	49	56	32	100	57	100	100	100	100	100	22	100	100	100	80	100
ehdA	Time (h)	P1	P2	P3	P4	P5	P6	P7	P8	P9	P10	P11	P12	P13	P14	P15	P16	P17	P18	P19	P20	P21	P22	P23	P24	P25	P26
	0	8	7	13	79	0	11	11	34	35	6	14	22	74	5	7	5	27	30	100	10	7	18	82	7	9	22
	2	12	24	24	13	23	27	0	27	22	10	16	15	46	11	23	56	42	51	60	10	37	21	21	51	100	29
	6	12	51	34	100	87	51	100	30	98	23	65	50	91	27	33	57	46	59	75	16	20	31	100	100	57	0
	8	37	76	56	14	32	87	40	30	90	72	76	31	100	29	70	48	47	98	74	23	38	47	37	44	48	57
	11	100	100	100	52	100	100	88	100	100	100	100	100	40	100	100	100	100	100	94	100	100	100	25	32	86	100
spiA	Time (h)	P1	P2	P3	P4	P5	P6	P7	P8	P9	P10	P11	P12	P13	P14	P15	P16	P17	P18	P19	P20	P21	P22	P23	P24	P25	P26
	0	50	15	10	15	35	20	4	23	18	2	47	37	33	18	6	10	0	2	31	13	6	64	29	11	0	10
	2	55	4	51	1	0	93	8	8	13	9	9	2	32	23	8	82	11	20	32	17	33	66	29	19	33	39
	6	80	19	27	100	71	32	100	33	46	21	100	100	100	24	11	85	83	93	50	28	20	42	32	37	51	84
	8	67	51	30	1	100	85	91	13	40	100	53	37	75	29	50	70	71	95	89	31	33	66	84	72	82	84
	11	100	100	100	12	62	100	70	100	100	92	67	33	96	100	100	100	100	100	100	100	100	100	100	100	100	100
cdcA	Time (h)	P1	P2	P3	P4	P5	P6	P7	P8	P9	P10	P11	P12	P13	P14	P15	P16	P17	P18	P19	P20	P21	P22	P23	P24	P25	P26
	0	19	4	13	38	7	4	9	32	27	15	13	14	18	16	2	9	4	5	28	34	58	8	13	2	13	29
	2	7	9	49	24	3	100	52	23	32	12	22	19	13	31	100	36	8	12	79	21	100	2	19	4	19	73
	6	19	94	50	44	40	83	94	30	30	36	100	35	97	97	35	37	50	43	99	42	83	34	56	31	46	100
	8	14	85	38	100	42	75	100	35	55	77	70	85	92	77	97	98	51	94	100	59	79	64	71	87	24	80
	11	100	100	100	92	100	38	93	100	100	100	52	100	100	100	38	100	100	100	97	100	89	100	100	100	100	72
pakA	Time (h)	P1	P2	P3	P4	P5	P6	P7	P8	P9	P10	P11	P12	P13	P14	P15	P16	P17	P18	P19	P20	P21	P22	P23	P24	P25	P26
	0	10	25	21	7	5	5	19	6	33	20	21	6	16	6	6	10	7	16	9	17	59	6	6	1	6	35
	2	23	88	32	28	6	53	42	59	40	10	38	10	13	13	89	100	6	20	11	32	13	32	9	8	22	51
	6	21	75	49	22	46	83	39	41	100	47	98	100	11	37	87	75	92	75	31	37	66	100	100	34	53	51
	8	24	64	52	100	83	100	61	47	31	81	99	99	100	73	100	55	78	93	60	32	77	70	40	55	58	57
	11	100	100	100	46	100	87	100	100	0	100	100	61	98	100	87	62	100	100	100	100	100	90	42	100	100	100
gapA	Time (h)	P1	P2	P3	P4	P5	P6	P7	P8	P9	P10	P11	P12	P13	P14	P15	P16	P17	P18	P19	P20	P21	P22	P23	P24	P25	P26
	0	7	2	5	21	16	5	7	38	10	6	7	29	17	9	9	2	17	25	42	34	8	30	46	2	0	3
	2	7	1	4	41	11	13	26	8	9	11	36	22	18	79	11	14	33	30	41	30	9	79	80	27	23	8
	6	22	15	5	93	100	19	21	4	29	28	45	72	100	76	35	33	56	51	73	19	29	57	86	70	77	19
	8	20	45	18	76	52	74	35	100	57	85	72	100	98	86	55	96	74	100	58	27	36	92	93	100	77	79
	11	100	100	100	100	65	100	100	19	100	100	100	98	54	100	100	100	100	53	100	100	100	100	100	93	100	100
pldA	Time (h)	P1	P2	P3	P4	P5	P6	P7	P8	P9	P10	P11	P12	P13	P14	P15	P16	P17	P18	P19	P20	P21	P22	P23	P24	P25	P26
	0	6	6	9	1	1	0	5	35	7	3	5	13	31	1	0	2	5	0	9	2	20	6	13	20	4	17
	2	20	4	92	1	4	35	76	1	18	14	10	3	13	5	15	21	14	7	10	10	22	20	30	0	19	35
	6	41	80	80	40	100	100	64	100	22	28	80	41	66	15	23	75	100	48	24	36	59	38	100	68	27	44
	8	38	100	52	100	58	18	100	13	80	50	58	33	100	32	45	98	73	100	33	34	69	75	79	78	83	60
	11	100	98	100	95	86	13	60	7	100	100	100	100	98	100	100	100	52	85	100	100	100	100	87	100	100	100
ligA	Time (h)	P1	P2	P3	P4	P5	P6	P7	P8	P9	P10	P11	P12	P13	P14	P15	P16	P17	P18	P19	P20	P21	P22	P23	P24	P25	P26
	0	7	4	4	27	31	1	4	20	12	17	9	2	16	1	2	1	17	29	3	4	18	1	11	3	0	4
	2	6	11	10	96	24	16	10	11	11	8	7	13	30	9	10	22	11	22	8	4	5	40	46	7	7	14
	6	22	70	8	94	27	13	59	17	46	21	42	36	52	34	21	54	100	22	40	9	34	43	100	50	7	9
	8	41	100	47	98	18	19	74	33	58	68	79	57	66	71	66	79	34	24	83	43	52	52	95	76	16	16
	11	100	96	100	100	100	100	100	100	100	100	100	100	100	100	100	100	44	100	100	100	100	100	90	100	100	100
pwiA	Time (h)	P1	P2	P3	P4	P5	P6	P7	P8	P9	P10	P11	P12	P13	P14	P15	P16	P17	P18	P19	P20	P21	P22	P23	P24	P25	P26
	0	7	1	22	7	3	19	1	6	1	2	5	5	2	2	15	0	9	2	5	4	11	3	19	3	1	2
	2	0	1	9	1	1	4	3	0	5	0	4	5	1	7	17	53	0	54	13	3	24	9	3	24	5	10
	6	5	19	26	82	17	17	39	11	26	16	46	32	32	40	13	75	49	81	40	2	22	14	37	54	7	17
	8	13	68	78	80	37	21	30	23	56	96	100	51	63	67	87	100	64	100	72	0	89	7	45	68	29	45
	11	100	100	100	100	100	100	100	100	100	100	33	100	100	100	100	86	100	58	100	100	100	100	100	100	100	100
rgsA	Time (h)	P1	P2	P3	P4	P5	P6	P7	P8	P9	P10	P11	P12	P13	P14	P15	P16	P17	P18	P19	P20	P21	P22	P23	P24	P25	P26
	0	1	45	8	2	6	1	1	7	1	2	2	3	1	0	1	0	0	0	37	18	13	4	7	0	0	4
	2	1	44	50	0	3	8	2	10	2	1	3	8	2	11	6	9	20	7	22	27	8	17	8	2	10	8
	6	2	100	15	54	14	22	13	2	4	8	5	36	27	15	16	11	31	7	1	36	18	35	72	24	14	45
	8	3	29	18	64	14	15	17	4	14	35	21	46	32	23	29	23	54	65	24	47	25	56	92	84	19	71
	11	100	20	100	100	100	100	100	100	100	100	100	100	100	100	100	100	100	100	100	100	100	100	100	100	100	100
		6d, 5 min FR				6d, 15 min FR									7d, 20 min FR				7d, 30 min FR								
